# Two new species of genus *Limnias* from Thailand, with keys to congeners (Rotifera, Gnesiotrocha)

**DOI:** 10.3897/zookeys.787.28098

**Published:** 2018-10-02

**Authors:** Phuripong Meksuwan, Rapeepan Jaturapruek, Supiyanit Maiphae

**Affiliations:** 1 Zoology Laboratory, Science Program, Faculty of Science and Technology, Phuket Rajabhat University, Ratsada, Muang District 83000, Phuket, Thailand Phuket Rajabhat University Phuket Thailand; 2 Department of Zoology, Faculty of Science, Kasetsart University 10900, Bangkok Thailand Kasetsart University Bangkok Thailand

**Keywords:** corona, dorsal plate, horny process, SEM, sessile rotifers

## Abstract

Two new species and two morphological variant taxa of sessile rotifers found during a survey of Thai rotifers are reported upon. Living specimens were collected and identified from twelve sampling sites. The specimens were photographed, and prepared for SEMs of dorsal plates and trophi. Two new species of genus *Limnias* are recognized. *Limniaslenis***sp. n.** possesses a corona with a relatively shallow ventral sinus, and a dorsal plate without any projection, whereas *Limniasnovemceras***sp. n.** carries a corona with a deep and wide ventral sinus, and a dorsal plate with nine projections. Two morphological variants of *Limniasmelicerta* are discussed, which are designated as *L.melicerta* SH and *L.melicerta* LH on the basis of the length of the horns on their dorsal plates. Moreover, both a dichotomous key and a formula key are provide including all *Limnias* species known to date.

## Introduction

*Limnias* Schrank, 1803 is a cosmopolitan genus of sessile rotifers (Segers 2007). This taxon is recognized by a combination of four characteristics: (1) a corona with two lobed and a wide dorsal gap, (2) a pair of distinct ventral antenna, (3) presence of a stiff dorsal plate, and (4) a firm tube that the adults inhabit (see figure 1 of Wallace et al. 2018). At present, six valid names of congeners are known: *L.ceratophylli* Schrank, 1803, *L.melicerta* Weisse, 1848, *L.myriophylli* (Tatem, 1868), *L.shiawasseensis* Kellicott, 1888, *L.cornuella* Rousselet, 1889 and *L.nymphaea* Stenroos, 1898 (Jersabek et al. 2015; Wallace et al. 2018). In Thailand, two species of *Limnias* had to date been identified, *L.melicerta* and *L.ceratophylli* by Koste (1975) and Meksuwan et al. (2011). One easily recognized characteristic that separates species in the genus is the architecture of tube. For example, in *L.melicerta* the tube is composed of a series of rings stacked vertically forming a tube (Wright 1954). The tube of *L.ceratophylli* lacks rings. Moreover, besides the ringed tube, *L.melicerta* possesses a deep ventral sinus corona and dorsal plate with seven projections, while *L.ceratophylli* has a corona with a shallow ventral sinus and a dorsal plate without projection. Based on our survey of diversity of sessile rotifers in Thailand started in 2011, we recognized two taxa belonging to *Limnias* which have distinct characters that do not fit any of the known members. They are here described as new species. We also update the dichotomous and formula keys to all species of genus *Limnias* by Wallace et al. (2018).

## Materials and methods

### Collecting sessile rotifers

Specimens of *Limnias* species were collected from different localities in Thailand (Supplementary material 1). Collecting method for sessile rotifers was described by Edmondson (1944), Wallace (1977), and Meksuwan et al. (2011). Briefly, it includes moving live, aquatic plants into a container filled with water from the sampling site to a container without adding any anesthetics or other chemicals for preservation. In laboratory, plants were dissected into convenient sizes for manipulation and examination for presence of sessile rotifers. These were identified alive. We suggest filtering some source water through a 60-μm, mesh plankton net and adding this to the containers to provide phytoplankton and organic particles as food source for the rotifers. By using this mesh size, larger zooplankton that may hinder examination are removed. Samples may be held for several days by providing suitable conditions. For examination plasticine was used to form small supports at the four corners of a coverslip to prevent compression of specimens.

### Scanning electron microscopy (SEM)

**Dorsal plate.** Each fixed, contracted specimen in 95% ethyl alcohol is extracted from its tube, and placed into a small drop of distilled water on a piece of cover glass. The specimen is oriented dorsally and left until the water is completely evaporated. Dried specimens on cover glasses are coated with gold, followed by examination under SEM.

**Trophi.** A sorted specimen is placed into a drop of commercial bleach (7% NaOCl). The remaining trophi is picked up and rinsed several times in drops of distilled water on a piece of cover glass. Then, the trophi is air dried, coated with gold and examined under SEM. SEM photographs were processed by a FEI Quanta 400 SEM.

## Results

### Descriptions of new species

Classification of genus *Limnias* follows Segers (2002). We adhere to this view as it is supported by a number of molecular analysis that support Eurotatoria, as taxon consisting of subclass Bdelloidea and subclass Monogononta (e.g., Melone et al. 1998, Mark Welch 2000). Subclass Monogononta comprises superorder Pseudotrocha and superorder Gnesiotrocha. Genus *Limnias* is located in the Gnesiotrocha where the members lack a toe.

### Phylum Rotifera Cuvier, 1817

#### Class Eurotatoria De Ridder, 1957

##### Subclass Monogononta Plate, 1889

###### Superorder Gnesiotrocha Kutikova, 1970

####### Order Flosculariaceae Harring, 1913

######## Family Flosculariidae Ehrenberg, 1838

######### Genus *Limnias* Schrank, 1803

########## Limnias lenis sp. n.

Taxon classificationAnimaliaFlosculariaceaeFlosculariidae

http://zoobank.org/8D2467C6-7CF9-4F0D-8ABA-52AC36E4E176

########### Material examined.

***Holotype*.** A contracted female in a mounted slide deposited in Princess Maha Chakri Sirindhorn Natural History Museum (PSUNHM), Prince of Songkla University, Songkhla, Thailand: PSUZC-PK5PM4–1. ***Paratypes*.** Two females in PSUNHM: PSUZC-PK5PM4-2–3; two females in Zoology Laboratory, Science Program, Faculty of Science and Technology, Phuket Rajabhat University, Phuket, Thailand: PKRU-RF2-1–2. In total, seven specimens were examined.

########### Type locality.

Jik peat swamp, Phuket Province, Thailand: 8°8.683'N, 98°17.983'E. Size of the peat swamp is about 230×140 meters. Individuals of *L.lenis* sp. n. were found attached to roots of Water hyacinth (*Eichhorniacrassipes* (C.Mart.) Solms). November 15^th^, 2015.

########### Etymology.

The species name is an adjective, derived from the Latin “lenis”, meaning “soft, smooth, gentle” and refers to the smooth surface of its dorsal plate.

########### Diagnosis.

**Figure 1.**
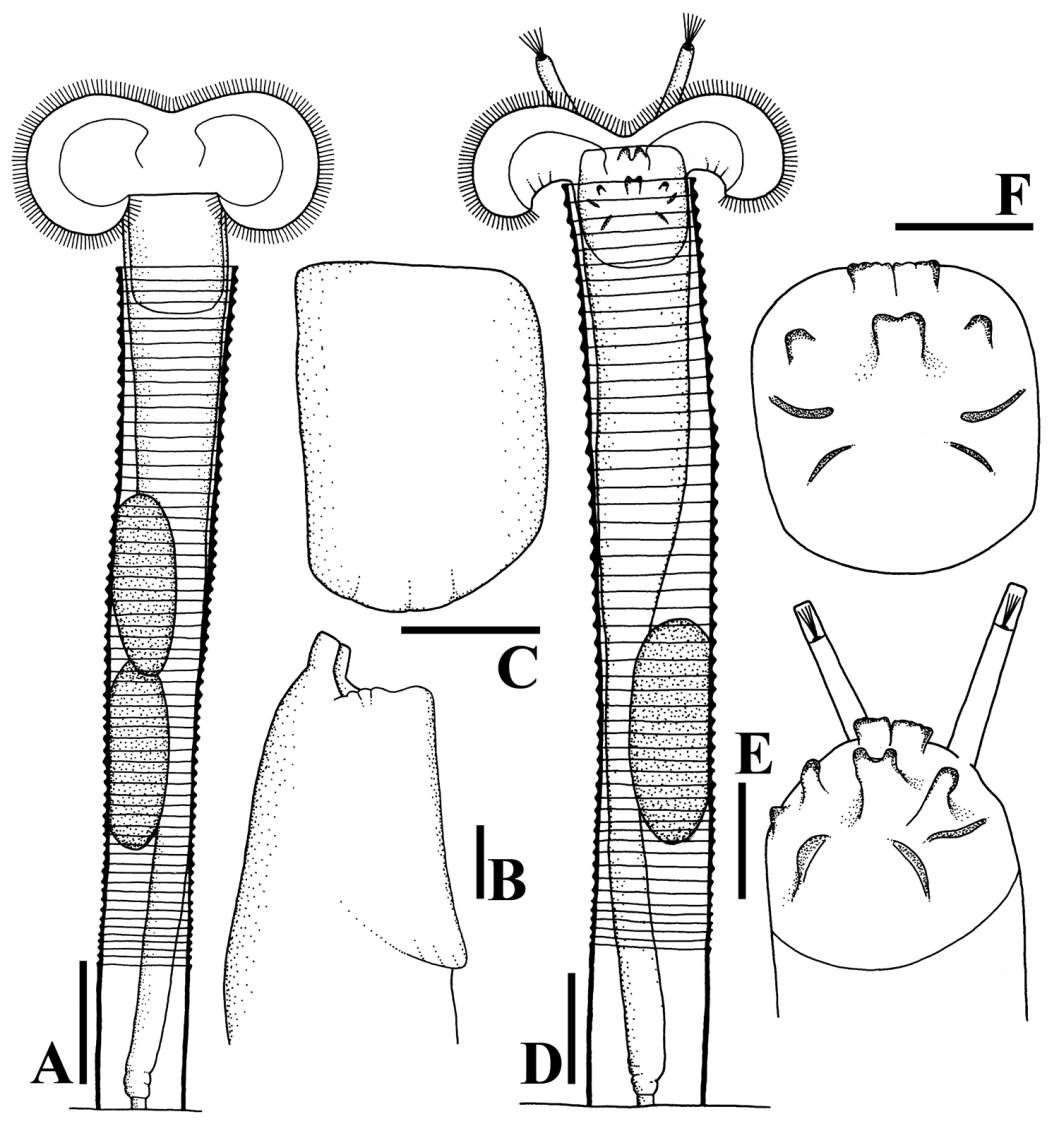
Line drawings of habitus and dorsal plate. **A–C***Limniaslenis* sp. n. **D–F***Limniasnovemceras* sp. n. Scale bars: 50 µm **(A, D)**; 25 µm **(B, C, E, F)**.

The species is unique by its corona having a relatively shallow ventral sinus compared to other taxa, and by its smooth dorsal plate, without any projection (Figs 1A–C, 3A–C, 7A).

**Figure 2.**
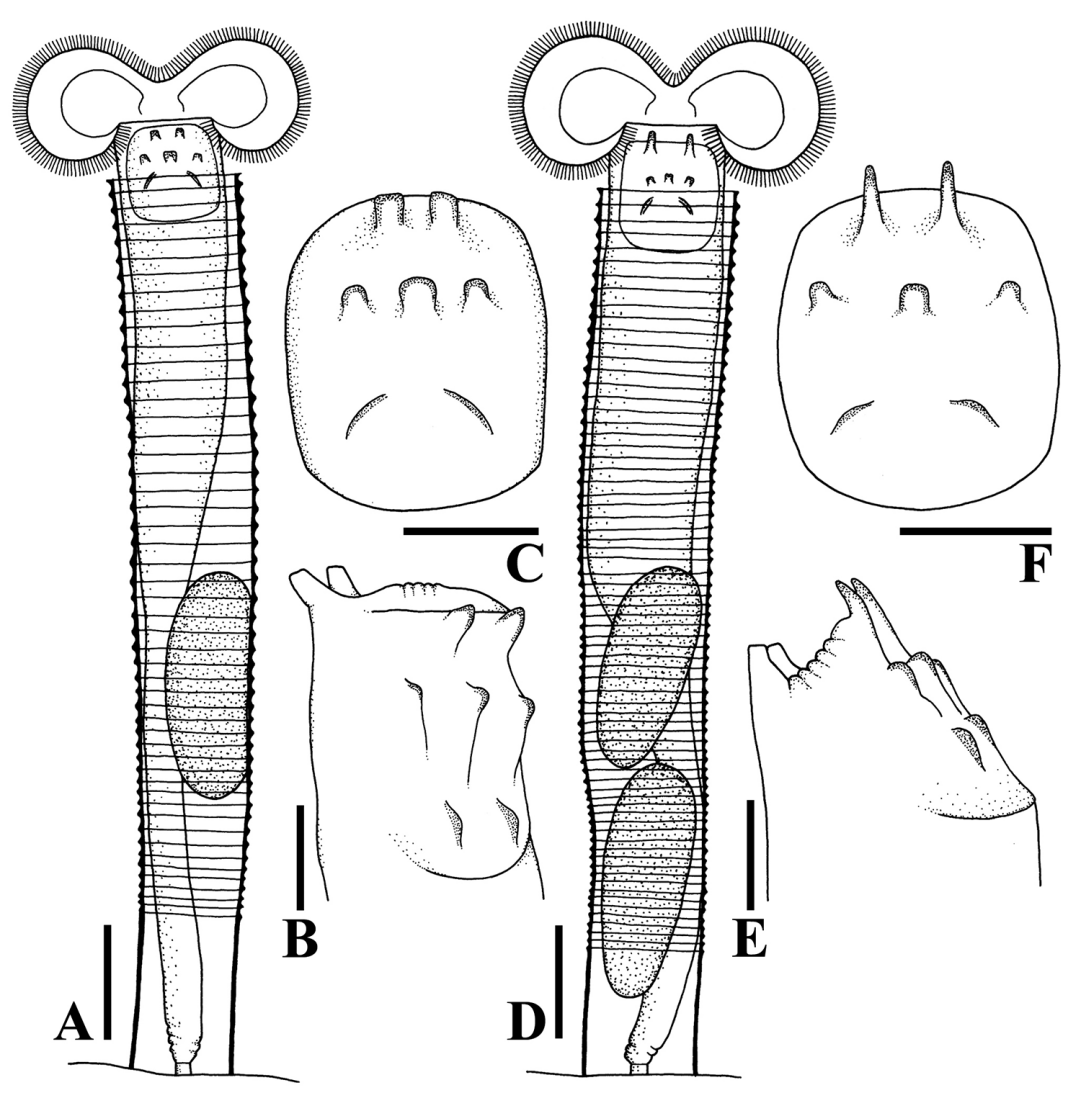
Line drawings of habitus and dorsal plate. **A–C***Limniasmelicerta* SH **D–F***Limniasmelicerta* LH. Scale bars: 50 µm **(A, D)**; 25 µm **(B, C, E, F)**.

**Figure 3.**
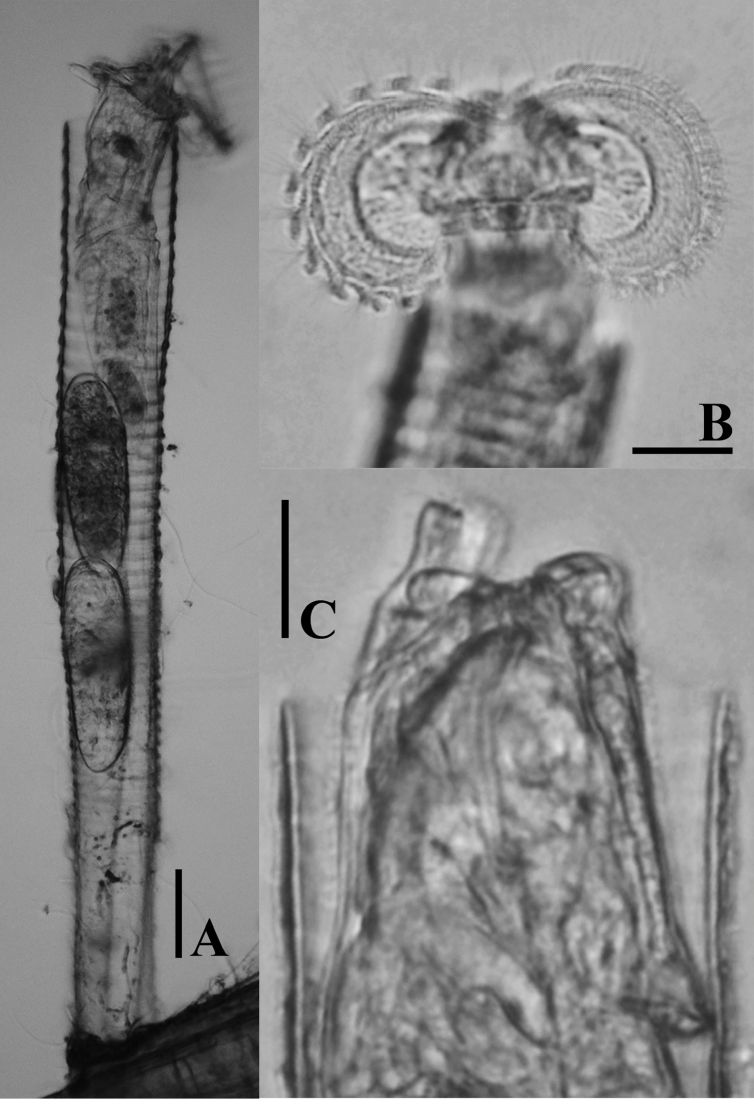
LM photographs of *Limniaslenis* sp. n. **A** habitus **B** corona **C** dorsal plate. Scale bars: 50 µm **(A)**; 25 µm **(B, C)**.

########### Description.

Tube ringed, proximal part smooth, usually transparent. Trunk slender, tapers into slender foot with short stalk (Figs 1A, 3A). Corona two-lobed, twice as wide as trunk, lobes nearly circular, ventral sinus shallow, dorsal gap large, as wide as trunk or nearly so (Figs 1A, 3B). Two short ventral antennae not extending beyond the corona. Dorsal plate smooth, without any projection. Dorsal antenna aperture situated one-fifth of dorsal plate length from the anterior margin (Figure 7A). Trophi malleoramate, symmetrical. Left and right proximal unci teeth: 3/3, distal teeth: 17/17 (Figure 8A). Rami apophyses equal. Manubria crescent-shaped with three chambers. Corona width: 98–110, corona height: 49–58, depth of ventral sinus: 7–12, width of dorsal gap: 33–38, length of lateral antennae: 9–14 (in µm).

########### Distribution.

The species is known only from its type locality.

########## Limnias novemceras sp. n.

Taxon classificationAnimaliaFlosculariaceaeFlosculariidae

http://zoobank.org/A54A2E46-DE6B-4F0C-A401-B9A69F248CE8

########### Material examined.

**Holotype.** A contracted female in a mounted slide was deposited in Princess Maha Chakri Sirindhorn Natural History Museum (PSUNHM), Prince of Songkla University, Songkhla, Thailand: PSUZC-PK5PM3-1. **Paratypes.** Two females in PSUNHM: PSUZC-PK5PM3-2–3; four females in Zoology Laboratory, Science Program, Faculty of Science and Technology, Phuket Rajabhat University, Phuket, Thailand: PKRU-RF1-1–4. In total, nine specimens were examined.

########### Type locality.

A stream in Krabi Province, Thailand: 8°12.687'N, 98°46.899'E. Individuals of *L.novemceras*, sp. n. were found on leaves of *Hydrillaverticillata* (L.f.) Royle growing in littoral area of the stream. June 2^nd^, 2011.

########### Etymology.

The species name is a substantive, and refers to the number of projections (nine, fromthe Latin *novem*, and horn, from the Greek *ceras*) on the dorsal plate.

########### Diagnosis.

**Figure 4.**
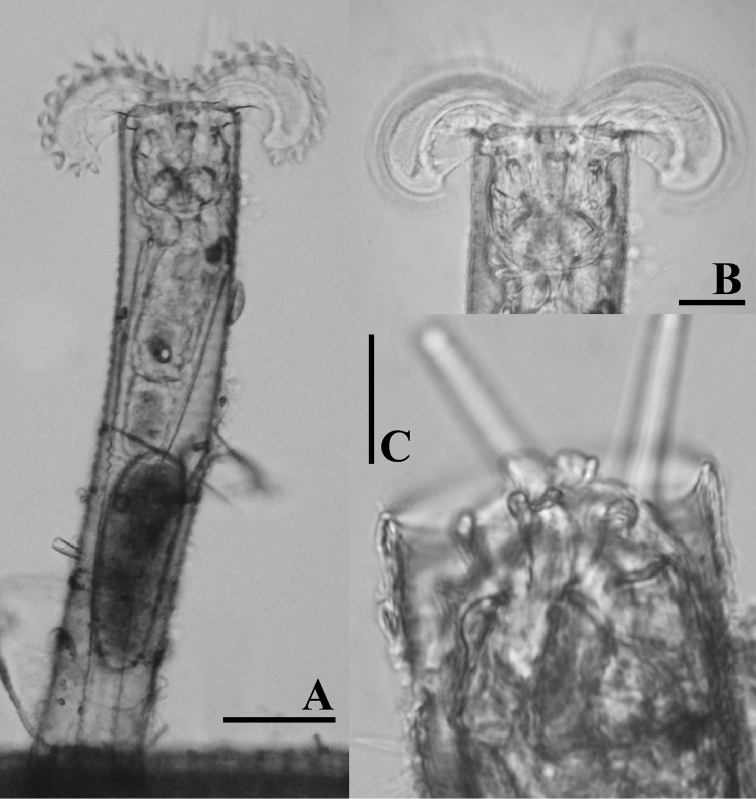
LM photographs of *Limniasnovemceras* sp. n. **A** habitus **B** corona **C** dorsal plate. Scale bars: 50 µm (**A**); 25 µm (**B, C**).

*Limniasnovemceras* sp. n. is easily recognized by its dorsal corona gap being much wider than the tube diameter, by its long ventral antennae that reach beyond the fully extended corona, and by its dorsal plate carrying nine projections. In addition, this species never raises its corona far beyond the tube opening (Figs 1D–F, 4A–C, 7B).

########### Description.

Tube ringed, transparent, proximal part smooth, transparent. Trunk slender, tapers into slender foot with short stalk (Figs 1D, 4A). Corona two-lobed, more than twice as wide as trunk, ventral sinus depth between one third and one half of corona height, dorsal gap nearly 1.5 times as wide as trunk width (Figs 1D, 4B). Two long ventral antennae extending beyond the extended corona. Dorsal plate stiff, with nine projections, upper row a pair of short and broad projections, middle row with a central, distally bifid projection, two lateral rounded projections and, slightly more distally, two low and broad, ridges, lower row a pair of oblique, rounded, low projections, these rounded triangular with straight inner margin in SEM preparation. Dorsal antenna aperture situated at one fifth of the dorsal plate length from the anterior margin (Figure 7B). Trophi malleoramate, symmetrical. Left and right proximal unci teeth: 3/3, distal teeth: 16–17/16–17 (Figure 8B). Rami apophyses equal. Manubria crescent-shaped with three chambers. Corona width: 114–126, corona height: 41–47, depth of ventral sinus: 11–16, width of dorsal gap: 74–80, length of lateral antennae: 43–44 (in µm).

########### Distribution.

The species is known only from its type locality.

######### Keys to species of genus *Limnias*

We constructed dichotomous and formula keys of all known *Limnias* species based on investigation of four species, including the two new species, observed in Thailand. In addition to the four species, we reevaluate the identity of *L.cornuella*, *L.myriophylli*, *L.nymphaea* and *L.shiawasseensis*, based on original publications, the most recent revision of the group by Wallace et al. (2018), and illustrations available in the Rotifer World Catalog (Jersabek and Leitner 2013). We recognize that number of dorsal plate projections, length of ventral antennae, and tube structure are useful characters for species identification in genus *Limnias*.

######### Dichotomous key

**Table:** 

1	Tube without ringed structure, usually covered with debris	**2**
–	Tube with ringed structure, clear or slightly colored (yellow or brownish) and sometimes covered with debris	**4**
2	Dorsal plate with seven projections	*** L. shiawasseensis ***
–	Dorsal plate without projection	**3**
3	Ventral antennae short, not reaching beyond fully extended corona	*** L. ceratophylli ***
–	Ventral antennae longer than fully extended corona	*** L. myriophylli ***
4	Dorsal plate without projection	***L.lenis* sp. n.**
–	Dorsal plate with projections	**5**
5	Ventral antennae short	**6**
–	Ventral antennae long	**7**
6	Seven projections on dorsal plate	*** L. melicerta ***
–	Fourteen projections on dorsal plate	*** L. nymphaea ***
7	Four projections on dorsal plate, tube curved or twisted	*** L. cornuella ***
–	Nine projections on dorsal plate, corona lobes separated by a very wide dorsal gap (nearly twice of truck width)	***L.novemceras* sp. n.**

######### Formula key to species

1 Ring tube: (a) absent; (b) present (Figs 1A, 3A)

**Figure 5.**
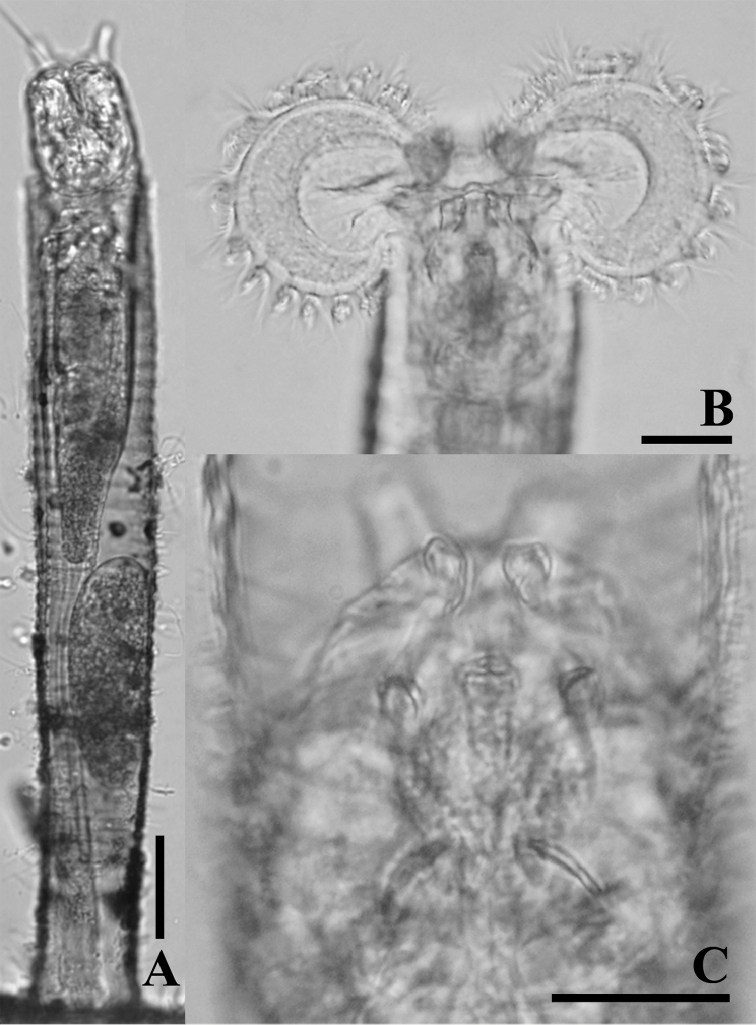
LM photographs of *Limniasmelicerta* SH. **A** habitus **B** corona **C** dorsal plate. Scale bars: 50 µm **(A)**; 25 µm **(B, C)**.

2 Dorsal plate projections: (a) absent (Figs 3C, 7A); (b) four; (c) seven (Figs 5C, 7C); (d) nine (Figs 1F, 7B); (e) fourteen

3 Ventral antennae length relative to fully extended corona: (a) shorter; (b) longer

######### Species included in the formula key:

*L.ceratophylli*: 1a, 2a, 3a

*L.myriophylli*: 1a, 2a, 3b

*L.shiawasseensis*: 1a, 2c, 3b

*L.cornuella*: 1b, 2b, 3b

*L.melicerta*: 1b, 2c, 3a

*L.nymphaea*: 1b, 2e, 3a

*L.lenis* sp. n.: 1b, 2a, 3a

*L.novemceras* sp. n.: 1b, 2d, 3b

## Discussion

**Figure 6.**
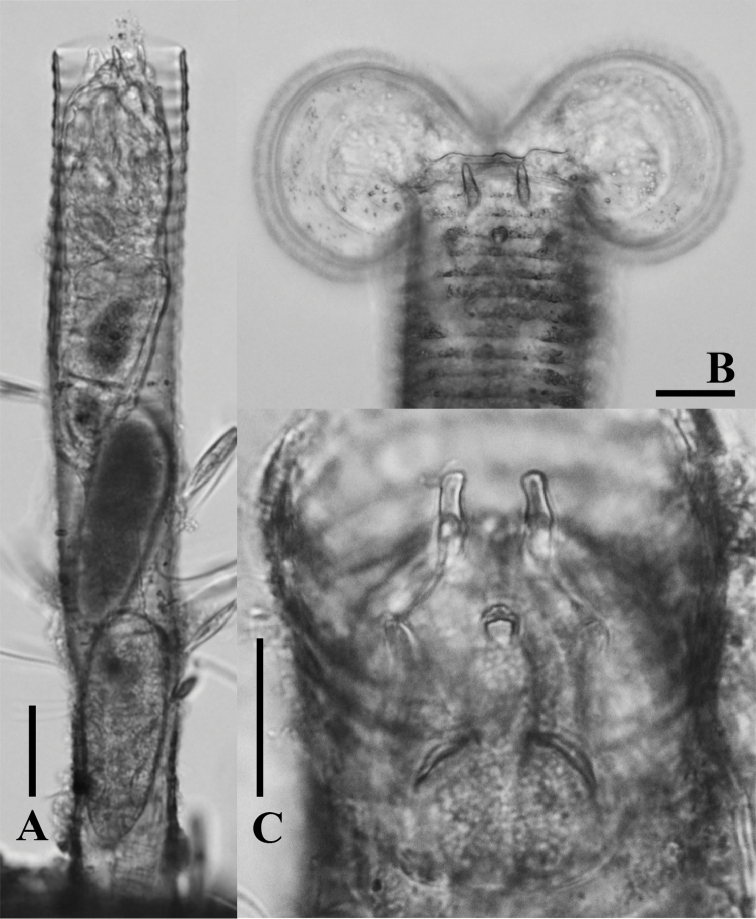
LM photographs of *Limniasmelicerta* LH. **A** habitus **B** corona **C** dorsal plate. Scale bars: 50 µm (**A)**; 25 µm **(B, C)**.

The most distinctive features of genus *Limnias* are the bilobed corona, and apparently, the presence of a rigid dorsal plate. While feeding this plate is located in the neck region on dorsal side of the body. When the rotifers are disturbed, however, they retract the corona into the tube and the dorsal plate is moved into an antero-dorsal position such that the horn-like projections are exposed towards the opening of the tube (Figs 4C, 6A). The dorsal plate would seem to act as a lid to the tube, and, hence, a possible defensive mechanism against predators, and may therefore be of adaptive value.

Our keys to species of *Limnias*, as well as those by Wallace et al. (2018), start off with tube having either ringed (annulated) or smooth appearance, albeit that it may be covered by debris. However, R.L. Wallace (personal communication) informed that there is uncertainty on tube structure of *L.shiawasseensis*. The photomicrograph of a mounted specimen deposited in the Academy of Natural Sciences of Drexel University identified as *L.shiawasseensis* (ANSP No.486) appears to show what looks like rings (Jersabek and Leitner 2013). To the contrary, the original description of the species by Kellicott, 1888 reads as follows: “The tube is normal in form, clear below and covered above by dark floccose; the surface is not smooth nor yet annulate, but beset with transverse, parallel rows of minute raised points which serve to hold the floccose which the animal packs against the tube by its dorsal processes”. We concur with Wallace that the original description should be privileged over the identification of the ANSP specimen. Therefore, we included *L.shiawasseensis* in our keys as a species lacking a ringed tube, until prove to the contrary.

At present, four species of *Limnias* rotifers have been recorded from Thailand: *L.ceratophylli*, *L.melicerta*, *L.lenis* sp. n. and *L.novemceras* sp. n. However, we found two additional, clearly distinguishable morphological variants of *L.melicerta* that both fit the original description of this species. Because no type specimens are known to exist for *L.melicerta* (see Jersabek et al. 2015), and as the original description of the species lacks the necessary detail, we are unable to determine whether, or which of the two Thai variants corresponds with the species. The two forms differ in the length and shape of the upper pair of projections (horns) on their dorsal plate; we refer to them as *L.melicerta* SH (Figs 2A–C, 5) and *L.melicerta* LH (Figs 2D–F, 6). SH has shorter horns (6.45±0.98 SD; n = 8) and was found in sampling sites S4, S5, S6 and S10, while the LH has longer horns (13.94±1.37 SD; n = 3), and was found in S5, S6, S10, S11 and S12 (Supplementary material 1). We were unable to find any additional characteristic on which we could base a separation; thus, molecular data will need to be analyzed to confirm whether the two taxa are separate species.

**Figure 7.**
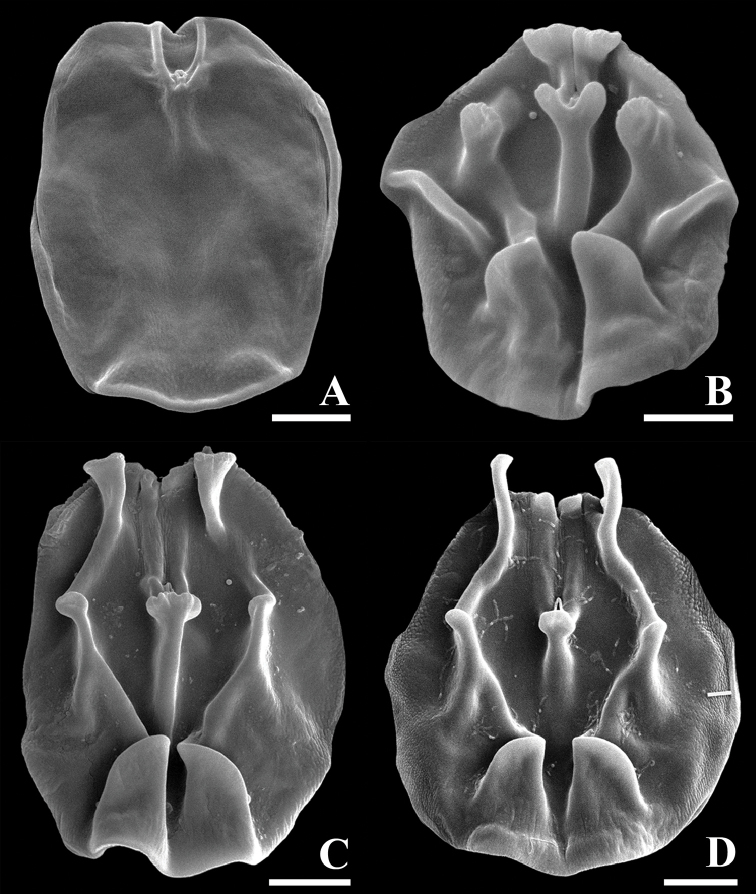
SEM photographs of dorsal plate (dorsal view). **A***Limniaslenis* sp. n. **B***Limniasnovemceras* sp. n. **C***Limniasmelicerta* SH **D***Limniasmelicerta* LH. Scale bars: 10 µm.

**Figure 8.**
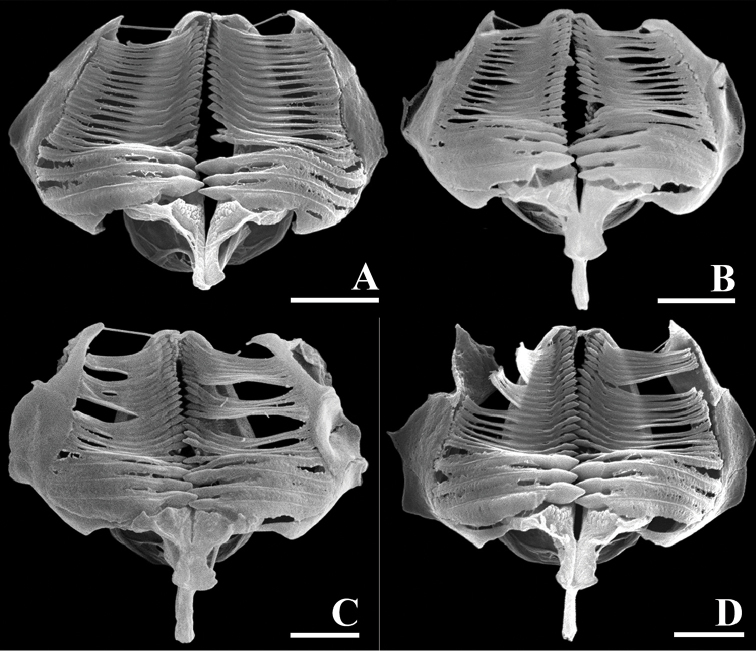
SEM photographs of trophi (frontal view). **A***Limniaslenis* sp. n. **B***Limniasnovemceras* sp. n. **C***Limniasmelicerta* SH **D***Limniasmelicerta* LH. Scale bars: 5 µm.

## Supplementary Material

XML Treatment for Limnias lenis

XML Treatment for Limnias novemceras

## Supplementary materials

Supplementary material 1 Sampling sites (S1–S12) in Thailand with GPS coordinates. Data type: occurence This is an open access article distributed under the terms of the Creative Commons Attribution License (CC BY 4.0), which permits unrestricted use, distribution, and reproduction in any medium, provided the original author and source are credited.Phuripong Meksuwan, Rapeepan Jaturapruek, Supiyanit Maiphae
