# Supplementary material for: Two new species of genus Limnias from Thailand, with keys to congeners (Rotifera, Gnesiotrocha)
Source: Zookeys. 2018 Oct 2;(787):1–15. doi: 10.3897/zookeys.787.28098 (PMC6177515; doi:10.3897/zookeys.787.28098)
Supplement: Supplementary material 1 — Sampling sites (S1–S12) in Thailand with GPS coordinates. [file zookeys-787-001-s001.pdf]

**Supplementary Table 1.** Sampling sites (S1–S12) in Thailand with GPS coordinates.

| Code | Province | GPS coordinate |
|---|---|---|
| S1 | Chiang Rai | 20° 15.209'N, 100° 2.854'E |
| S2 | Phayao | 19° 11.493'N, 99° 51.525'E |
| S3 | Phichit | 16° 25.626'N, 100° 20.006'E |
| S4 | Udon Thani | 17° 12.820'N, 103° 2.231'E |
| S5 | Bueng Kan | 18° 19.033'N, 103° 40.740'E |
| S6 | Bueng Kan | 18° 1.419'N, 104° 0.780'E |
| S7 | Ranong | 9° 47.643'N, 98° 35.580'E |
| S8 | Krabi | 8° 12.687'N, 98° 46.899'E |
| S9 | Phuket | 8° 8.683'N, 98° 17.983'E |
| S10 | Phatthalung | 7° 47.626'N, 100° 8.377'E |
| S11 | Trang | 7° 31.789'N, 99° 45.280'E |
| S12 | Satun | 6° 44.557'N, 100° 2.575'E |
